# An Unusual Case of *Campylobacter jejuni* Gastroenteritis Presenting with Acute Reversible Encephalopathy in an Immunocompetent Host

**DOI:** 10.1155/2020/9603428

**Published:** 2020-07-04

**Authors:** Irma Huayanay, Leonardo Pozo, Salman Bangash, Denisse Ramirez, Luis Rosas, Renzo Arauco Brown

**Affiliations:** ^1^School of Medicine, The University of Texas Rio Grande Valley, Edinburg, Texas, USA; ^2^Internal Medicine Residency Program, University of Texas Rio Grande Valley, Edinburg, Texas, USA; ^3^Doctors Hospital at Renaissance, Edinburg, Texas, USA; ^4^South Texas Infectious Disease Consultants, McAllen, Texas, USA; ^5^Pulmonary and Sleep Center of the Valley, McAllen, Texas, USA

## Abstract

*Campylobacter jejuni* gastroenteritis is the most frequent organism associated with acute infectious diarrhea worldwide. The clinical presentation involves fever, diarrhea, rigors, and myalgias. Other extraintestinal symptoms that have been described involve delirium and other neurological complications, and the most well-known is Guillain-Barré, where there is cross-reactivity between the gastrointestinal tract and the brain. Despite previously described multiple neurological complications, there is a lack of clinical data on the association of *Campylobacter*-related gastroenteritis with acute encephalopathy in immunocompetent patients. The type of population, immunocompetent stage, and unfamiliarity with the clinical presentation makes this a challenging diagnosis for clinicians. We report a case of *Campylobacter* gastroenteritis associated with acute encephalopathy in an immunocompetent patient.

## 1. Introduction

*Campylobacter jejuni* (*C. jejuni*) is the most frequent organism associated with acute bacterial gastroenteritis around the world [1]. The high incidence of *Campylobacter* diarrhea, as well as its duration and possible complications, makes it highly important from a socioeconomic perspective [2]. It is estimated to cause 1.3 million human illnesses every year with 96 million cases in 2010 in the United States [3]. The clinical presentation is characterized mainly by abdominal pain and diarrhea. A prodrome phase characterized by fever, rigors, myalgias, and occasional delirium has been described [2]. The most well-known postinfectious neurological complications are Guillain-Barré syndrome (GBS) and Miller-Fisher syndrome (MFS) [4]. Furthermore, worldwide the incidence is 1 to 2 in 100,000 and 1 to 2 in 1,000,000, for GBS and MFS, respectively [5]. Nearly 30–40% of GBS patients had *C. jejuni* infection 2 weeks prior to onset of neurological symptoms [6]. Other less frequent neurological complications include Bickerstaff's brainstem encephalitis, acute transverse myelitis, and acute disseminated encephalomyelitis (ADEM) [4, 7–9]. We present a case of *Campylobacter* gastroenteritis presenting with associated encephalopathy in an immunocompetent patient.

## 2. Case

A twenty-five-year-old female with unremarkable past medical history was brought to the emergency department due to progressive altered mental status. Per family report, the patient was in her usual state of health until one week before this admission when she started to experience abdominal pain and diarrhea. The patient's mother described episodes of anxiety and unusual behavior-associated confusion specially the day before admission.

On the day of admission, the patient sought medical attention at an outpatient clinic. While in the office, she became confused and later she experienced a witnessed seizure. The patient received a dose of lorazepam prior to arrival at the hospital. On arrival to the emergency department, she was found unresponsive with a Glasgow Coma Scale of six. She was afebrile and tachycardic with a heart rate of 115 beats per minute. The rest of her vital signs were within normal limits.

Her physical exam was remarkable for generalized decreased muscle tone and no clear focal deficits. The patient was intubated for airway protection. She was given a loading dose of levetiracetam and was admitted to the neurosciences intensive care unit.

Her initial laboratory tests showed respiratory alkalosis with an elevated white blood cell count at 11.6 th/*μ*l with 76.5% of neutrophils. Her complete metabolic panel showed hypokalemia. Her urine toxicology, serum alcohol levels, and urine analysis were unremarkable. *C*-reactive protein was 3.3 mg/dL, and her lactic acid was 1.2 mmol/L. Blood tests are summarized in Table 1.

**Figure 1:**
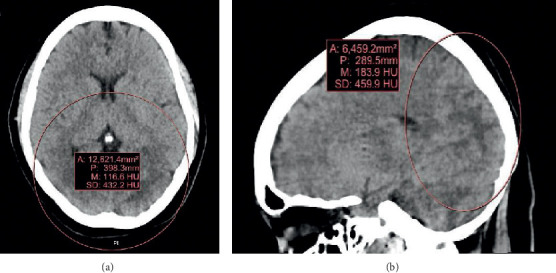
CT head without contrast showing increased low density in the left occipital white matter.

A brain computed tomography (CT) without contrast showed slightly increased low density in the left occipital white matter compared to the right which increased the suspicion of posterior reversible encephalopathy syndrome (PRES), and these images are shown in Figure 1. Herpes simplex virus (HSV) encephalitis and autoimmune epilepsy were also considered in the differential diagnosis. The brain CT with perfusion was negative for stroke. Chest X-ray showed atelectatic changes in the right upper lobe. A CT of the abdomen and pelvis was negative for ovarian teratoma. The patient was started on acyclovir for suspected herpetic encephalitis.

A brain magnetic resonance (MRI) was negative for findings supporting the diagnosis of PRES. The electroencephalogram showed no evidence of seizure activity or epileptiform discharges. Lumbar puncture was performed, and the cerebrospinal fluid (CSF) had a clear appearance. The opening pressure was normal. The CSF glucose was elevated at 91 units. The CSF white blood cell count was 3/*μ*L. Further studies on CSF for ruling out meningitis were done, and they included *Cryptococcus neoformans/gattii*, *Cytomegalovirus*, *Enterovirus* CSF, *E. coli* K1, *Hemophilus influenzae*, Human *Parechovirus*, *Listeria monocytogenes*, *Neisseria meningitidis*, *Streptococcus agalactiae*, *Streptococcus pneumoniae*, varicella-zoster, and herpes simplex virus 1, 2, and 6, which were negative; details are shown in Table 2.

The infectious disease team was consulted, and doxycycline was added due to the high prevalence of typhus in the area. Febrile agglutinins, West Nile virus, and fungal serologies were also negative (Table 1).

CSF studies for autoimmune epilepsy, including N-methyl-D-aspartate (NMDA) receptor antibodies, glutamic acid decarboxylase (GAD-65) antibodies, and voltage-gated potassium channel (VGKC) antibodies, were negative for autoimmune epilepsy, the more detailed studies in Table 2. Human immunodeficiency virus was negative. Autoimmune panel was negative for antineutrophil cytoplasmic antibodies (ANCAs), antinuclear antibody (ANA), and double stranded DNA (dsDNA) antibodies.

Due to persistent diarrhea during the initial day of admission, a gastrointestinal infectious panel was ordered and was positive for *C. jejuni* (Table 3).

Based on the clinical scenario of a patient with extraintestinal complications of *Campylobacter* infection, it was decided to start azithromycin 500 mg per mouth for at least 10 days. The patient showed significant improvement after the first dose and recovered over the next few days prior to discharge.

## 3. Discussion

We are describing a case of *Campylobacter jejuni* gastroenteritis associated with acute encephalopathy in an immunocompetent adult. The complex initial presentation with neurological symptoms makes this clinical scenario a diagnostic challenge due to the unfamiliarity of clinicians with this disease.

*Campylobacter* species belongs to a distinct group of specialized Gram-negative bacteria designated as rRNA superfamily VI [10]. The most important species that can cause human disease are *C. jejuni* and *C. coli* [11]. Campylobacters invade the intestinal epithelium using flagella, high molecular weight plasmids, superficial adhesins, and chemotactic factors [12].

Patients infected with *C. jejuni* gastroenteritis experience acute watery or bloody diarrhea, fever, weight loss, and cramps that last six days on average [11]. Besides the gastrointestinal local infection, *Campylobacter* species also can cause a range of other clinical manifestations or postinfectious immune disorders [13].

While infection with *C. jejuni* can occur in patients of all ages, this infection is more prevalent in toddlers and young adults than in other age groups [14]. Despite most of the neurologic complications associated with *Campylobacter* enteritis have been historically linked to the pediatric population, some of these manifestations have been also described in adults [9].

Our patient had an unusual presentation of *C. jejuni* gastroenteritis associated with acute encephalopathy. Based on her initial symptoms, she was treated with acyclovir for the suspicion of HSV encephalitis. Moreover, the combination of seizures in association with the abnormalities noted in the mentioned neuroimaging raised the initial suspicion for PRES and autoimmune epilepsy. These diagnoses were ruled out with a brain MRI and a CSF autoimmune panel, respectively [15–17].

Other autoimmune systemic diseases including lupus and vasculitis were ruled out with negative ANCA, ANA, and dsDNA. We also considered ADEM following a *C. jejuni* gastroenteritis in our differential diagnosis. Nonetheless, the MRI did not show increased T2-signal intensity in the white matter which is a characteristic neuroimaging finding in ADEM [4]. Furthermore, patients suffering ADEM only improve with high-dose steroids that our patient never received [4].

Based on Rio Grande Valley demographics with high prevalence of murine typhus in the area, the patient was treated empirically with doxycycline until her agglutinins were negative [18]. In this clinical case, the neurological and gastrointestinal symptoms rapidly resolved once the patient was started on azithromycin for *Campylobacter* gastroenteritis. Azithromycin is the antibiotic of choice, has good coverage for *Campylobacter jejuni*, shortens the duration of symptoms, and decreases resistance compared to fluoroquinolones [19, 20].

At this present time, the exact pathophysiology of *Campylobacter* gastroenteritis associated with encephalopathy is unknown, but it is possible that mechanisms involving antibody cross-reactivity may play a role [9]. For example, it is well known that bidirectional signaling exists between the gastrointestinal tract and the brain in Guillain-Barré syndrome after a *C. jejuni* infection, where the outer membrane structures of *Campylobacter* containing sialic acid resemble those seen in the human gangliosides [9].This brings the topic of gut-brain axis, which explains that everything is interconnected: the gut can influence the brain, the brain can influence the gut, and they both can influence and be influenced by the immune system [21].

There are only a few cases of *Campylobacter* gastroenteritis with encephalopathy in immunocompetent population, and one of them went through a similar analysis process of differential diagnoses, obtained similar workup as in our patient based on the neurological symptoms, age of the patient, immunocompetent status, and severity of symptoms onset, with clinical improvement after starting antibiotics [9].

Due to the low prevalence of *Campylobacter*-associated encephalopathy in adults, we do not recommend assuming that mental status changes in a patient presenting with *Campylobacter* gastroenteritis are necessarily associated with the infection. A proper and well-directed workup should be completed to rule out other more frequent and emergent neurological diseases such as bacterial meningitis or vascular disease. We would recommend considering *Campylobacter*-associated encephalopathy in the differential diagnosis of diarrhea associated with mental status changes.

## Figures and Tables

**Table 1:** Blood studies.

	Blood analysis	Result	Reference range
Complete blood count	White blood cell	11.6	(4.8–10.9) th/*μ*L
	Percent neutrophils	76.5	(49.0–77.6) %
	Percent lymphocytes	17.7	(11.8–40.8) %
	Percent monocytes	5	(3.70–11.80) %
	Percent eosinophils	0.2	(0–4.8) %
	Percent basophils	0.3	(0–1.3) %
	Percent immature granulocytes	0.3	(0–1) %
	Hemoglobin	12.6	(10.8–14.7) gm/dL
	Hematocrit	36.8	(32.2–42.9) %
	Mean corpuscular volume	87.4	(84–96) fL
	Mean corpuscular hemoglobin	29.9	(28.3–32.3) pg
	Mean corpuscular hemoglobin concentration	34.2	(29.58–33.75) gm/dL
	Platelet count	212	(146–388) th/*μ*L
	Red blood cell distribution width CV	12	(12.20–15.80) %
	Red blood cell distribution width SD	38.6	(38.93–51.44) fL

Complete metabolic panel	Sodium	136	(132–143) mmol/L
	Potassium	3.3	(3.5–5.1) mmol/L
	Chloride	105	(98–107) mmol/L
	Carbon dioxide	23	(21–31) mmol/L
	Creatinine	0.7	(0.6–1.2) mg/dL
	BUN	12	(7–25) mg/dL
	Calcium	8.8	(8.6–10.3) mg/dL
	Glucose	119	(70–113) mg/dL
	Ammonia	30	(16–53) umol/L
	AST	17	(13–39) IU/L
	ALT	15	(7–52) IU/L
	Alkaline phosphatase	73	(34–104) IU/L
	Albumin	4.1	(3.7–4.9) gm/dL
	Total bilirubin	0.3	(0.2–1.2) mg/dL

Coagulation studies	PT	12.9	(10.6–13) second
	INR	1.11	(0.90–1.10) INR
	PTT	22.8	(24–36.7) second

Others	Sedimentation rate	46	2–30 mm/hr
	C-reactive protein	3.3	<1 mg/dL
	Lactic acid	1.2	(0.5–1.99) mmol/L
	Alcohol serum	<10.00	<10.00 mg/dL
	Alcohol serum calculation	<0.01	<0.01 gm/dL

Serology	ANCA screen^*∗*^	Negative	Negative
	dsDNA	1.5	<10 IU/mL
	Symphony ANA screen	0.1	<0.7 ratio
	Symphony ANA	0.2	<0.7 ratio
	Complement C3	156	(87–200) mg/dL
	Complement C4	26	(19–52) mg/dL

Infectious studies	HIV antibodies	Nonreactive	Nonreactive
	*Aspergillus flavus* antibody	Negative	Negative
	*Aspergillus niger* antibody	Negative	Negative
	*Aspergillus fumigatus* antibody	Negative	Negative
	*Coccidioides* antibody	Negative	Negative
	Histoplasma antibody	Negative	Negative
	Blastomycosis antibody	Negative	Negative
	Febrile agglutinin panel^*∗*^	Negative	Negative
	Respiratory viral panel PCR^*∗*^	Negative	Negative

^*∗*^ANCA screen: p-ANCA, c-ANCA, and atypical p-ANCA. ^*∗*^Febrile agglutinin panel: P-OX19, POX2, P-OXK, and *Brucella*. ^*∗*^Respiratory viral panel includes adenovirus, coronavirus, influenza A, influenza B, human metapneumovirus, parainfluenza, respiratory syncytial virus, rhinovirus, *Enterovirus*, *Bordetella pertussis*, *Chlamydophila pneumoniae*, and *Mycoplasma pneumoniae*.

**Table 2:** CSF studies.

	Variable	Result	Reference range
General analysis	CSF appearance	Clear	Clear
	CSF WBC	3	0–5/*μ*L
	CSF RBC	0	0/*μ*L
	CSF glucose	91	40–70 mg/dL
	CSF total protein	26	15–45 mg/dL

Infectious studies	CSF *Cryptococcus neoformans/gattii* antigen	Not detected	Not detected
	CSF *Cytomegalovirus*	Not detected	Not detected
	CSF *Enterovirus*	Not detected	Not detected
	CSF *Escherichia coli* K1	Not detected	Not detected
	CSF *Hemophilus influenzae*	Not detected	Not detected
	CSF herpes simplex virus 1	Not detected	Not detected
	CSF herpes simplex virus 2	Not detected	Not detected
	CSF human herpes virus 6	Not detected	Not detected
	CSF human *Parechovirus*	Not detected	Not detected
	CSF *Listeria monocytogenes*	Not detected	Not detected
	*Neisseria meningitidis*	Not detected	Not detected
	*Streptococcus agalactiae*	Not detected	Not detected
	*Streptococcus pneumoniae*	Not detected	Not detected
	Varicella-zoster virus	Not detected	Not detected
	CSF West Nile IgM	<0.90	<0.90
	CSF culture	No growth	No growth

Autoimmune studies	CSF VGKC antibody	<20	<20 pmol/L
	CSF NMDA receptor antibody	Negative	Negative
	CSF NMDAR1 antibody, CBA	Negative	Negative
	CSF AMPAR1 antibody, CBA	Negative	Negative
	CSF AMPAR2 antibody, CBA	Negative	Negative
	GABABR antibody, CBA	Negative	Negative
	LGI1 antibody, CBA	Negative	Negative
	CASPR2 antibody, CBA	Negative	Negative
	GAD-65	<5	<5 IU/mL

VGKC: voltage-gated potassium channel; NMDA: N-methyl-D-aspartate; AMPAR: alpha-amino-3-hydroxy-5-methyl-4-isoxazolepropionate receptor; GABA: gamma aminobutyric acid B receptor; LGI1: leucine-rich glioma-inactivated 1; CASPR2: contactin-associated protein 2; GAD: glutamic acid decarboxylase.

**Table 3:** Stool studies.

	Variable	Result	Reference range
Gastrointestinal analysis	*Campylobacter*	Detected	Not detected
	*Clostridium difficile*	Not detected	Not detected
	*Plesiomonas shigelloides*	Not detected	Not detected
	*Salmonella*	Not detected	Not detected
	*Yersinia enterocolitica*	Not detected	Not detected
	*Vibrio*	Not detected	Not detected
	*Vibrio cholerae*	Not detected	Not detected
	Enteroaggregative *E. coli*	Not detected	Not detected
	Enteropathogenic *E. coli*	Not detected	Not detected
	Enterotoxigenic *E. coli*	Not detected	Not detected
	Shiga-like toxin-producing *E. coli*	Not detected	Not detected
	*E. coli* O157	Not detected	Not detected
	*Shigella*/enteroinvasive *E. coli*	Not detected	Not detected
	*Cryptosporidium*	Not detected	Not detected
	*Cyclospora cayetanensis*	Not detected	Not detected
	*Entamoeba histolytica*	Not detected	Not detected
	*Giardia lamblia*	Not detected	Not detected
	Adenovirus	Not detected	Not detected
	Astrovirus	Not detected	Not detected
	Norovirus	Not detected	Not detected
	Rotavirus	Not detected	Not detected
	Sapovirus	Not detected	Not detected
